# Microarray data analysis to identify miRNA biomarkers and construct the lncRNA-miRNA-mRNA network in lung adenocarcinoma

**DOI:** 10.1097/MD.0000000000030393

**Published:** 2022-09-09

**Authors:** Yongan Song, Leonardo Kelava, Lu Zhang, István Kiss

**Affiliations:** a Department of Public Health Medicine, University of Pécs Medical School, Szigeti str 12, Pécs 7624, Hungary; b Department of Thermophysiology, Institute for Translational Medicine, Medical School, University of Pécs, Szigeti str 12, Pécs 7624, Hungary; c Department of Health Science, Doctoral School of Health Science, University of Pécs, Vasvári Pál utca 4, Pécs 7622, Hungary.

**Keywords:** bioinformatics, GEO, lung adenocarcinoma, miRNA, overall survival, TCGA

## Abstract

MicroRNAs (miRNAs), regulatory noncoding RNAs, are involved in gene regulation and may play a role in cancer development. The aim of this study was to identify miRNAs involved in lung adenocarcinoma (LUAD) using bioinformatics analysis. MiRNA (GSE135918), mRNA (GSE136043) and lncRNA (GSE130779) microarray datasets were downloaded from the Gene Expression Omnibus (GEO) database to identify differentially expressed miRNAs (DEMis), mRNAs (DEMs), and lncRNA (DELs) in LUAD. We used DEMs for functional enrichment analysis. MiRNA expression quantification from The Cancer Genome Atlas (TCGA) was used to validate DEMis. LncBase Predicted v.2, Targetscan, and MiRBase were used to predict lncRNAs and mRNAs. The LUAD data in TCGA were used for overall survival (OS) analysis. We screened the downregulation of 8 DEMis and upregulation of 6 DEMis, and found that 70 signal pathways changed. We chose 3 relevant signaling pathways in lung cancer development, WNT, PI3K-Akt, and Notch, and scanned for mRNAs involved in them that are potential targets of these miRNAs. Then a lncRNA-miRNA-mRNA network was constructed. We also found 7 miRNAs that were associated with poor OS in LUAD. Low expression level of hsa-miR-30a was highly associated with poor OS in LUAD (*P* < .001) and the target genes of hsa-miR-30a-3p were abundant in the Wnt and AKT signaling pathways. In addition, our results reported for the first time that hsa-miR-3944 and hsa-miR-3652 were highly expressed in LUAD. And the high expression level of hsa-miR-3944 was associated with poor OS (*P* < .05). Hsa-miR-30a-3p may suppress the occurrence and progression of lung cancer through Wnt and AKT signaling pathways and become a good biomarker in LUAD. Hsa-miR-3944 and hsa-miR-3652 may serve as new biomarkers in LUAD.

## 1. Introduction

Lung cancer has been a major public health problem and is the leading cause of cancer deaths in the past few decades worldwide.^[1,2]^ Histologically, lung cancer can be divided into small cell lung cancer (SCLC) and non–small cell lung cancer (NSCLC). The latter comprises >80% of lung cancer cases and is subdivided into adenocarcinoma, squamous cell carcinoma, and large-cell carcinoma.^[3]^ Lung adenocarcinoma (LUAD) is the most common form of lung cancer, accounting for approximately 40% of all cases.^[4,5]^ Most LUADs have poor prognosis.^[6]^ The main reason is the difficulty in early diagnosis and the lack of effective treatment.^[7]^ Therefore, it is urgent to find biomarkers related to the prognosis of lung cancer patients, which may improve early diagnosis and contribute to the development of gene-targeted therapy.^[8]^

MicroRNAs (miRNAs) are small noncoding RNAs that interfere with mRNA translation through complementary base pairing to the 3′ untranslated region (UTR) of the target mRNA, leading to either mRNA degradation or inhibition of translation.^[9–11]^ Evidence shows that miRNAs have a variety of cellular regulatory effects, and some miRNAs function as oncogenes or tumor suppressor genes.^[12]^ Long noncoding RNAs (lncRNAs) are noncoding RNAs with length of 200 to 10,000 base pairs (bp).^[13]^ LncRNAs do not code for proteins, but rather regulate target gene expression at transcriptional and posttranscriptional levels.^[14]^ However, the clear role of lncRNA in tumors still needs further research. According to the competitive endogenous RNA (ceRNA) hypothesis,^[15–17]^ lncRNAs can have a sponge effect on miRNAs and weaken the influence of miRNAs on mRNAs. In addition, studies have shown that the networks of lncRNAs, miRNAs, and mRNAs play an important role in the pathogenesis and progression of cancer.^[16,18]^ Nevertheless, the study of large-scale samples in LUAD is not common. Therefore, screening of miRNAs that are relevant in LUAD and building a ceRNA network with miRNAs as the core is very important for the early diagnosis and treatment of LUAD patients.^[19,20]^ In the present study, we have analyzed the expression of genes (lncRNAs, miRNAs, and mRNAs) in LUAD gene expression profiles taken from the GEO database. In addition, we have established a ceRNA network in LUAD through bioinformatics methods in order to find new potential targets for cancer therapy.

The use of high-throughput microarrays for expression profiling has become a widely used technology; it can be used to measure the expression of thousands of genes at once and to identify new cancer biomarkers.^[21]^ In this study, we reported a comprehensive analysis of miRNA, mRNA, and lncRNA expression by reanalyzing the public data sets from GSE135918, GSE136043, and GSE130779. Compared to control, differentially expressed miRNAs (DEMis), differentially expressed mRNAs (DEMs), and differentially expressed lncRNAs (DELs) were identified in LUAD samples. We tried to predict the interactions between DEMis and DEMs, and then performed functional enrichment analysis to construct miRNA-gene regulatory networks and competitive endogenous RNA (ceRNA) networks. Through comprehensive bioinformatics analysis, we expected to find new therapeutic targets and biomarkers for LUAD.

## 2. Methods

### 2.1 Microarray data

Gene expression data sets were obtained from the GEO repository (https://www.ncbi.nlm.nih.gov/geo/), which is a public database containing gene expression data from high-throughput experiments.^[22]^ The expression record sets of human miRNAs and mRNAs, containing expression profiles of lung cancer tissue and adjacent tissue of the same 5 LUAD patients, were obtained from NCBI GEO (GSE135918, GSE136043). The lncRNA microarray data of 8 LUAD patients were downloaded from NCBI GEO (GSE130779), which also includes record of lung cancer tissues and adjacent tissues. Data from the GEO database do not require ethics committee approval.

### 2.2 Data preprocessing and screening of differential expression

The GEOquery package of the R platform was used to download the miRNA, mRNA, and lncRNA data, and the data were imported into the R statistical environment. The limma package was used^[23]^ for data preprocessing, including extraction of expression matrix, clinical information, platform annotation files and deletion of missing data. For miRNA data, the results of nonhuman miRNA probes were removed, and the data were log_2_ converted. The lncRNA data were effectively processed using normalization and log_2_ conversion. The platform annotation information was obtained from the GEO database, and the chip probe IDs were converted into gene symbols. The expression matrix is divided into a cancer tissue group and an adjacent nontumor group. The limma package was used to calculate the *P* value of the difference in gene expression between the cancer tissue group and the adjacent nontumor group, and the differentially expressed miRNAs (DEMis), mRNAs (DEMs), and lncRNAs (DELs) were selected in turn.^[23]^ According to *P* value < .05 and log_2_ fold change (FC) > 1, DEMis, DEMs, and DELs were filtered out. In order to visualize DEMis, DEMs, and DELs, the FactoMineR, factoextra, ggplot2, ggplotify, and pheatmap packages in the R platform were used to draw principal component analysis (PCA) maps, volcano maps, and heat maps.

### 2.3 Functional enrichment analysis

We used the clusterProfiler package in the R platform to perform Gene Ontology (GO) biological processes term and Kyoto Encyclopedia of Genes and Genomes (KEGG) pathway analysis on DEMs data.^[24–26]^ The results of the functional enrichment analysis were used to analyze the changes in molecular biological functions of lung cancer tissues.

### 2.4 Prediction of target lncRNAs and mRNAs of DEMs

The interaction between lncRNAs and miRNAs is predicted by LncBase Predicted v.2 of DIANA Tools.^[27]^ By setting the threshold of interaction score to 0.8, the predicted lncRNAs of lncRNA-miRNA pairs were further filtered (the score ranges from 0 to 1), and the information of DELs-DEMis pairs was obtained. Next, the targeted mRNAs of DEMis were retrieved from MiRTarBase and Targetscan.^[28,29]^ Both miRNA reference databases are reliable. Through the prediction results of these 2 databases, we then got the information of the DEMis-DEMs pairs.

### 2.5 DEMis validation

The TCGA database provides a wealth of clinical information and miRNA expression quantification from a huge sample size. Therefore, the data set “TCGA-LUAD” was used to screen DEMis obtained from GSE135918. We obtained the file containing all TCGA-LUAD clinical information and miRNA expression quantification from the database (https://portal.gdc.cancer.gov/). In the R platform, the DESeq2 package was used to calculate the *P* value and log_2_ FC value of the gene expression difference between the tumor group and the normal group to verify DEMis results obtained in the GEO database.

### 2.6 Construction of ceRNA network

The DELs-DEMis-DEMs network was reconstructed by integrating the prediction results of DEMis’ target lncRNAs and mRNAs. The Cytoscape software was used to visualize the DELs-DEMis-DEMs network.

### 2.7 The survival analysis of DEMs

In order to explore whether the DEMs we selected are related to overall survival (OS) of the patients, the LUAD data in TCGA were used for analysis. Another 2 R packages, survival and survminer, were used to calculate the OS analysis for DEMs. We separated the patients into high and low groups and stratified the miRNA expression levels of cancer patients through the surv_categorize function in the R platform. In addition, the chart showed the hazard ratio with the 95% confidence interval (CI) and the *P* value.

## 3. Results

### 3.1 Identification of DEMis

**Figure 1.**
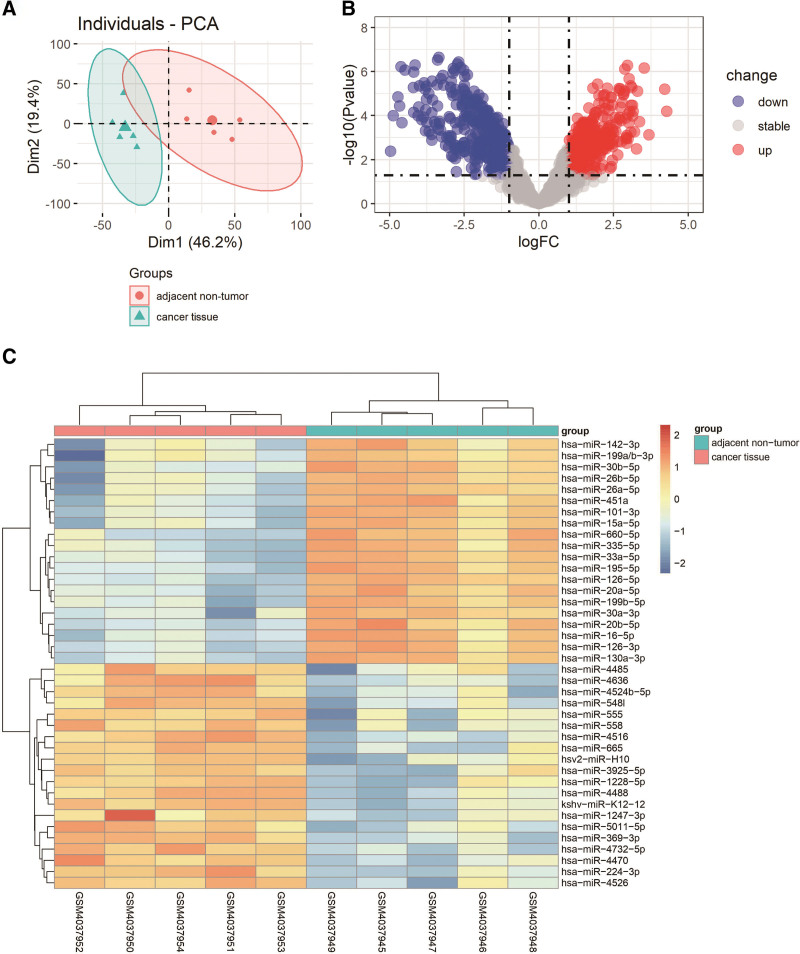
PCA, volcano plots, and heat map of DEMis. (A) PCA of DEMis. (B) Volcano plot of DEMis. (C) Heat map of DEMis. DEMis = differentially expressed miRNAs, PCA = principal component analysis.

We extracted miRNA data from GSE135918 for analysis. The expression levels of miRNA in lung cancer tissues and their adjacent tissues of 5 LUAD patients were studied. The cutoff for log FC of miRNA was 1, and the cutoff for *P* value was .05. A total of 272 upregulated miRNAs and 353 downregulated miRNAs were identified by removing the missing and duplicate data in the GSE135918. In Table 1, we provided the top 10 upregulated and downregulated ones including their symbol, log FC value, *P* value together with adjusted *P* value. Supplemental Digital Content (Appendix 1, Supplemental Digital Content, http://links.lww.com/MD/H189) is a complete file containing all miRNAs information. The principal component analysis (PCA) is shown in Figure 1A. In Figure 1B, the volcano map showed the miRNA distribution of -log_10_ (*P* value) and log_2_ FC correlation, while the heat map of DEMis is shown in Figure 1C, which directly shows the difference between the cancer tissue group and the adjacent nontumor group. It can be seen in Table 1 and Figure 1B that the downregulated miRNA is more significant than the upregulated miRNA in log FC.

**Table 1:** Top 10 upregulated and downregulated miRNAs in LUAD samples.

Top 10 upregulated miRNAs			Top 10 downregulated miRNAs		
Name	log FC	*P* value	Name	log FC	*P* value
hsa-miR-665	5.0413	3.70E-05	hsa-miR-20a-5p	-7.8193	4.37E-07
hsa-miR-548l	4.2852	6.55E-05	hsa-miR-33a-5p	-6.9344	3.49E-07
hsa-miR-4470	4.2097	6.50E-06	hsa-miR-126-5p	-6.7550	8.15E-09
hsa-miR-3925-5p	3.6836	0.000761	hsa-miR-126-3p	-6.6164	2.26E-07
hsa-miR-369-3p	3.5261	7.08E-07	hsa-miR-130a-3p	-6.5095	1.63E-07
hsa-miR-558	3.3719	0.000194	hsa-miR-199b-5p	-6.3718	2.77E-06
hsa-miR-4485	3.2740	0.003309	hsa-miR-20b-5p	-6.0270	4.26E-07
hsa-miR-1247-3p	3.1977	0.000545	hsa-miR-101-3p	-5.9727	1.10E-06
hsa-miR-4526	3.1544	6.36E-05	hsa-miR-195-5p	-5.7738	1.66E-07
hsa-miR-4636	3.1405	3.49E-05	hsa-miR-142-3p	-5.6201	0.001166

LUAD = lung adenocarcinoma.

### 3.2 Identification of DEMs

**Figure 2.**
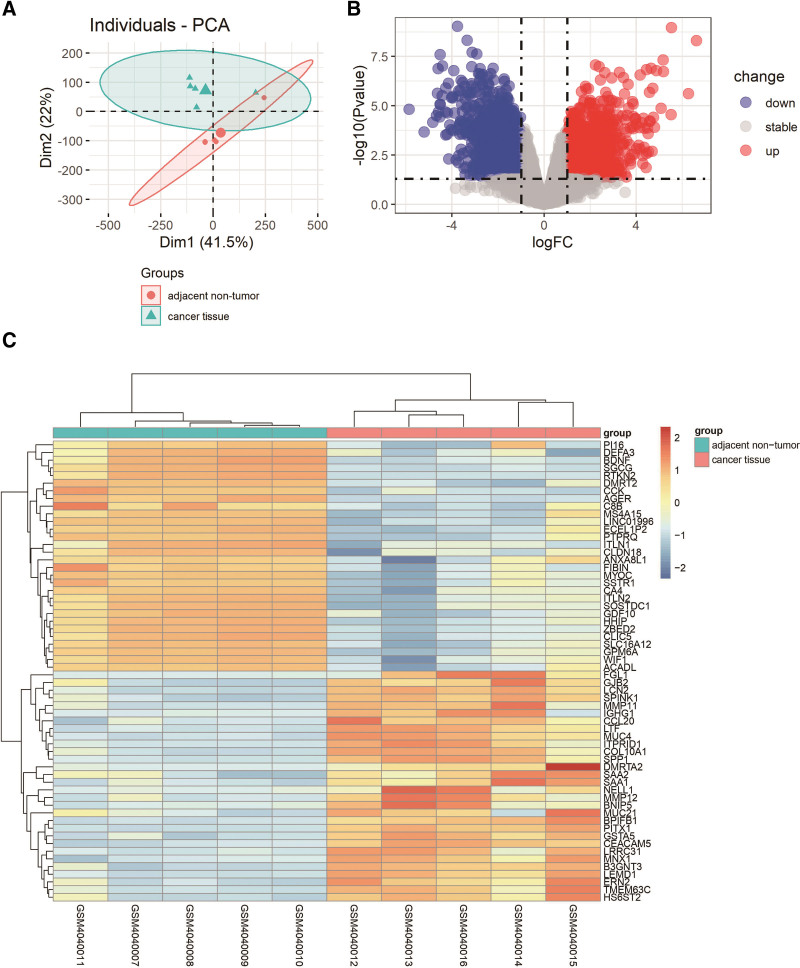
PCA, volcano plots, and heat map of DEMs. (A) PCA of DEMs. (B) Volcano plot of DEMs. (C) Heat map of DEMs. DEMs = differentially expressed mRNAs, PCA = principal component analysis.

The mRNA data were extracted from GSE136043, and the mRNA expression levels in lung cancer tissues and their adjacent tissues of 5 LUAD patients were analyzed. In order to explore the interaction between miRNA and mRNA more accurately, mRNA and miRNA data were from the same 5 patient samples. Like miRNA, the cutoff for log FC of mRNA was 1, and the cutoff for *P* value was .05. We deleted the missing and duplicate values in the GSE135918 data. The analysis results showed that 1659 mRNAs were upregulated and 1476 mRNAs were downregulated. Table 2 lists the top 10 upregulated and downregulated mRNAs, as well as their symbols, log FC, and *P* value. In addition, we show the complete DEMs file in Appendix 2, Supplemental Digital Content, http://links.lww.com/MD/H190. The PCA map is shown in Figure 2A. The volcano map (Fig. 2B), based on the data of all expression levels, vividly illustrates the distribution of all mRNAs on the correlation between -log_10_ (*P* value) and log_2_ FC. The heat map shown in Figure 2C shows the difference between the cancer tissue group and the adjacent nontumor group. We can see that the difference of upregulated mRNA was more significant than that of the downregulated mRNA (Table 2 and Fig. 2B).

**Table 2:** Top 10 upregulated and downregulated mRNAs in LUAD samples.

Top 10 upregulated mRNAs			Top 10 downregulated mRNAs		
Name	log FC	*P* value	Name	log FC	*P* value
CEACAM5	6.62529	4.96E-09	ITLN2	−5.8529	1.52E-05
SPP1	6.27753	2.43E-06	ITLN1	−5.2136	0.000214
PITX1	5.54022	1.08E-09	WIF1	−4.8259	6.86E-05
COL10A1	5.51926	2.86E-05	CA4	−4.6774	3.16E-05
SPINK1	5.18254	1.90E-07	LINC01996	−4.6352	2.34E-06
LCN2	5.17145	4.82E-08	CCK	−4.6056	1.29E-07
MNX1	5.08281	1.34E-05	SOSTDC1	−4.5878	2.10E-05
LEMD1	4.88463	1.64E-07	ECEL1P2	−4.5338	3.74E-05
ITPRID1	4.79101	0.000124	SLC16A12	−4.5151	8.51E-06
HS6ST2	4.75437	3.82E-05	PTPRQ	−4.5094	8.45E-06

LUAD = lung adenocarcinoma.

### 3.3 Identification of DELs

**Figure 3.**
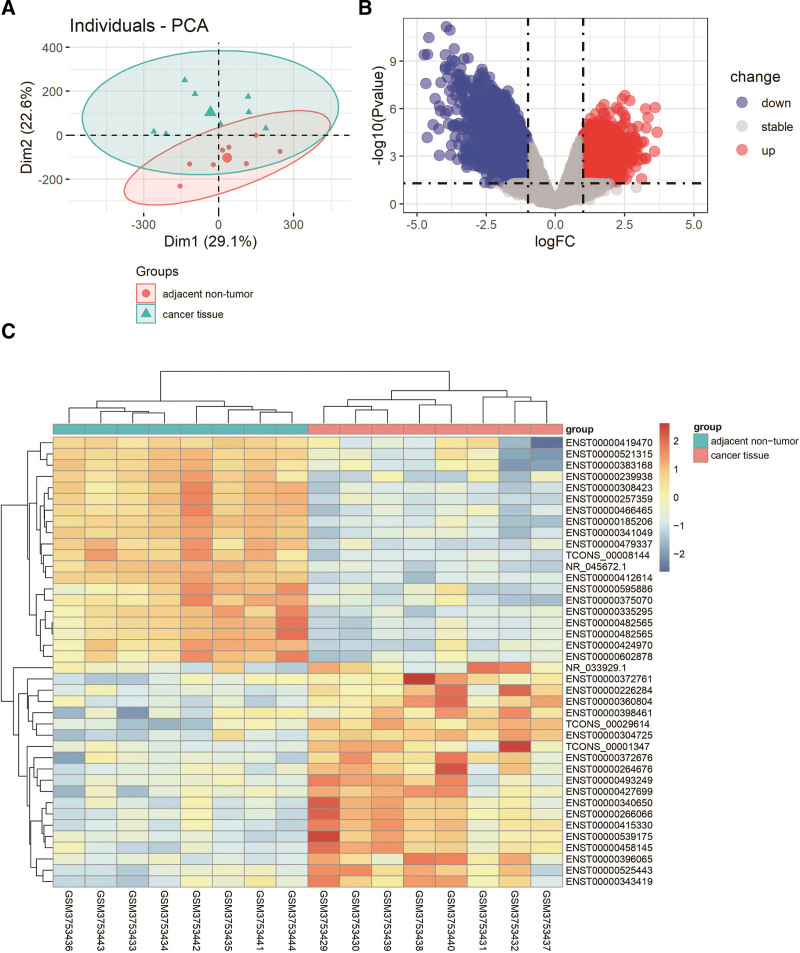
PCA, volcano plots, and heat map of DELs. (A) PCA of DELs. (B) Volcano plot of DELs. (C) Heat map of DELs. DELs = differentially expressed lncRNAs, PCA = principal component analysis.

The lncRNA data came from GSE130779. This study investigated the expression levels of lncRNA in the lung cancer tissues and their adjacent tissues of 8 LUAD patients. According to the screening criteria of miRNA and mRNA, the cutoff for log FC of lncRNA was 1, and the cutoff for *P* value was 0.05. We also deleted missing and duplicate values in the data. The limma package in the R platform was used to normalize and analyze the expression levels of all lncRNAs. The results showed that 4054 lncRNAs were upregulated and 5543 lncRNAs were downregulated. We provided the 10 most upregulated and downregulated lncRNAs in Table 3, including their symbols, log FC values, and *P* values. We also provided the complete DELs file in Appendix 3, Supplemental Digital Content, http://links.lww.com/MD/H191). Figure 3A is the PCA map. In Figure 3B, the volcano map shows the distribution of all DELs on the correlation between -log_10_ (*P* value) and log_2_ FC. The heat map of DELs is generated through the pheatmap package in the R platform. As shown in Figure 3C, the difference between the cancer tissue group and the adjacent nontumor group can be visually displayed. As we can see in Table 3 and Figure 3B, the downregulated lncRNA was more significant than the upregulated lncRNA.

**Table 3:** Top 10 upregulated and downregulated lncRNAs in LUAD samples.

Top 10 upregulated lncRNAs			Top 10 downregulated lncRNAs		
Name	log FC	*P* value	Name	log FC	*P* value
ENST00000266066	3.67741	3.10E-05	ENST00000185206	−4.7360	4.36E-06
ENST00000304725	3.61137	8.77E-07	ENST00000335295	−4.6792	5.04E-05
ENST00000427699	3.58888	0.000507	ENST00000595886.1	−4.6299	0.004522
ENST00000415330.2	3.34376	3.60E-06	NR_045672.1	−4.5730	8.32E-07
ENST00000360804	3.29212	1.64E-05	ENST00000521315	−4.4191	0.00172
TCONS_00001347	3.27638	0.001404	ENST00000482565	−4.3475	0.000205
TCONS_00029614	3.26817	1.05E-06	ENST00000383168	−4.2211	0.002354
ENST00000372761	3.25113	0.001324	ENST00000482565	−4.1887	0.000387
ENST00000398461.1	3.13564	0.001586	ENST00000419470	−4.1857	0.016868
NR_033929.1	3.12088	0.026358	ENST00000424970	−4.1613	0.00053

lncRNA = long noncoding RNA, LUAD = lung adenocarcinoma.

### 3.4 Functional enrichment analysis of DEMs

**Figure 4.**
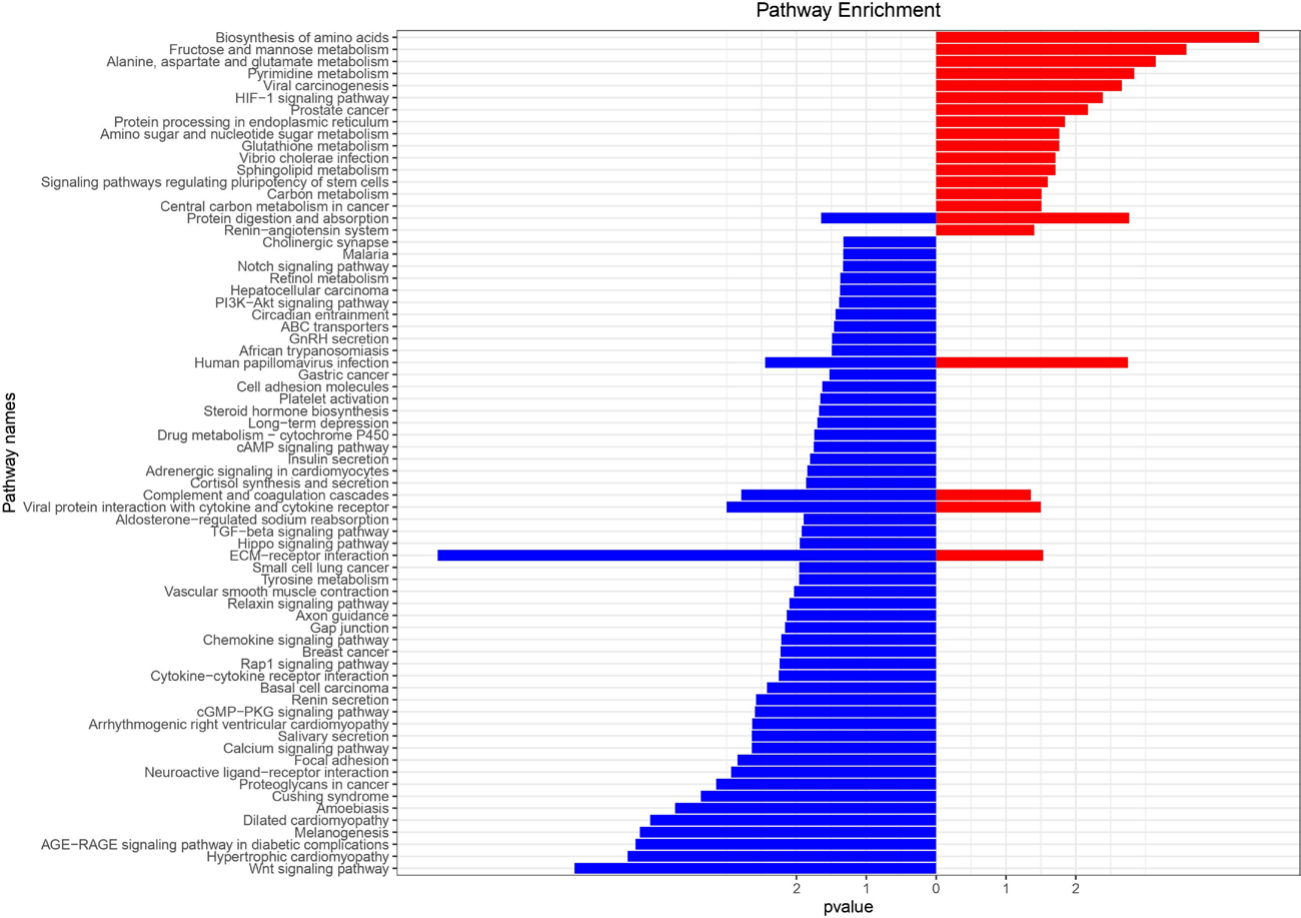
The enriched KEGG pathways of DEMs in cerebral infarction. DEMs = differentially expressed mRNAs, KEGG = Kyoto Encyclopedia of Genes and Genomes.

**Figure 5.**
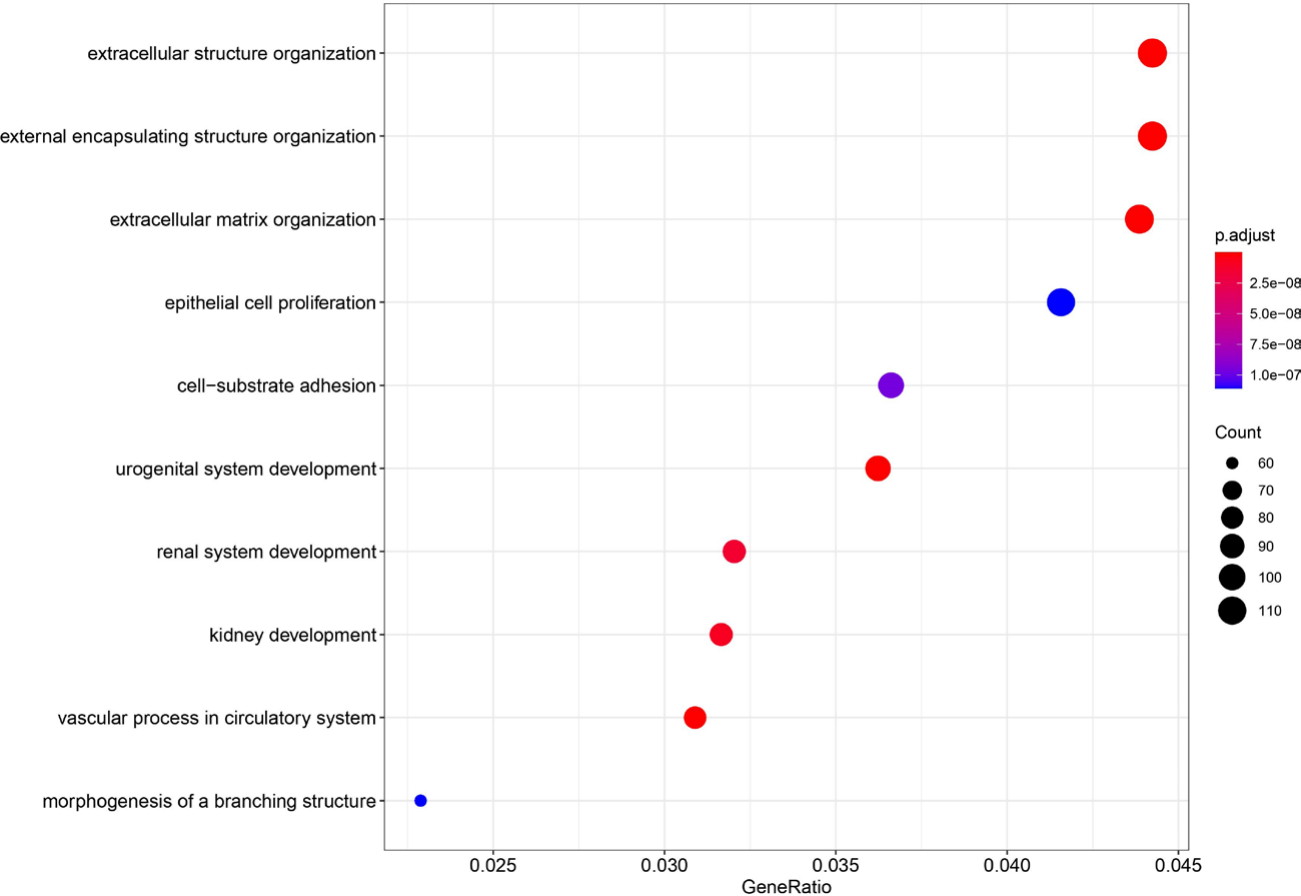
The first 10 enriched GO BP terms of DEMs in LUAD. DEMs = differentially expressed mRNAs, LUAD = lung adenocarcinoma.

The ClusterProfiler package in the R platform was used to perform KEGG and GO analysis on DEMs, elucidating the possible mechanisms involved in the development of LUAD. The threshold of *P* value was set to .05, yielding 70 KEGG signal pathways (Fig. 4). A complete file is provided in Appendix 4, Supplemental Digital Content, http://links.lww.com/MD/H192, and Appendix 5, Supplemental Digital Content, http://links.lww.com/MD/H193, including their GeneRatio, *P* value, geneID, and count. Among them, Wnt, PI3K-Akt, and Notch signaling pathways were implicated in the development of lung cancer. DEMs were used for GO biological process enrichment. We selected the results with *P* value < .01 and q-value < 0.01, and used the top ten results of GeneRatio to create a bubble chart (Fig. 5).

### 3.5 miRNA screening

The 50 upregulated and downregulated miRNAs with the most significant differential expression were selected and verified with the miRNA data in the TCGA database. All clinical information on miRNA expression quantification of LUAD (TCGA-LUAD) was downloaded from https://portal.gdc.cancer.gov/. The results showed that there were 567 LUAD samples, including 46 normal samples and 521 tumor samples. After comparison of TCGA data to our top 50 downregulated and top 50 upregulated miRNAs, we verified the downregulation of 8 DEMis (hsa-miR-101-3p, hsa-miR-195-5p, hsa-miR-30a-3p, hsa-miR-451a, hsa-miR-144-3p, hsa-miR-15b-5p, hsa-miR-193a-3p, hsa-miR-145-5p) and the upregulation of 6 DEMis (hsa-miR-665, hsa-miR-369-3p, hsa-miR-224-3p, hsa-miR-381, hsa-miR-3944-3p, hsa-miR-3652). Because these 14 miRNAs were differentially expressed in both GEO and TCGA databases, they had a high degree of credibility. In order to analyze whether the 14 differentially expressed miRNAs were related to the occurrence of lung cancer, we selected mRNAs related to Wnt signaling pathway, PI3K-Akt signaling pathway, and Notch signaling pathway from the target mRNA predicted by miRNA to explore the interactions between miRNA and these 3 signaling pathways.

### 3.6 Construction of ceRNA network

**Figure 6.**
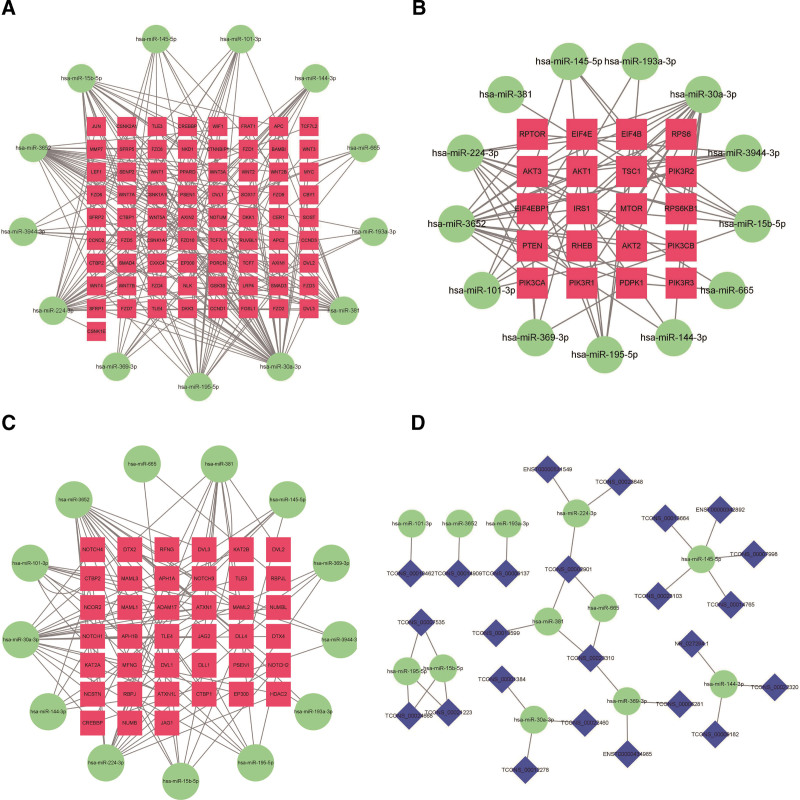
ceRNA regulatory network in LUAD. (A) DEMis mediated miRNA-mRNA (the key miRNAs in Wnt signaling pathway) regulatory network. (B) DEMis mediated miRNA-mRNA (the key miRNAs in PI3K-Akt signaling pathway) regulatory network. (C) DEMis mediated miRNA-mRNA (the key miRNAs in Notch signaling pathway) regulatory network. (D) DELs-DEMis regulatory network. ceRNA = competitive endogenous RNA, DELs = differentially expressed lncRNAs, DEMis = differentially expressed miRNAs.

Targetscan and MiRBase were used to predict the target genes of these 14 miRNAs we identified. We selected the key mRNAs in the aforementioned 3 signaling pathways from these target genes. These 14 miRNAs and these key mRNAs were used to construct a miRNA-mRNA regulatory network (Fig. 6A–C). Then LncBase Predicted v.2 of DIANA Tools was used to predict the interaction between lncRNAs and miRNAs. The DELs interacting with these 14 miRNAs were selected, and the lncRNA-miRNA regulatory network was constructed (Fig. 6D).

### 3.7 Survival analysis

**Figure 7.**
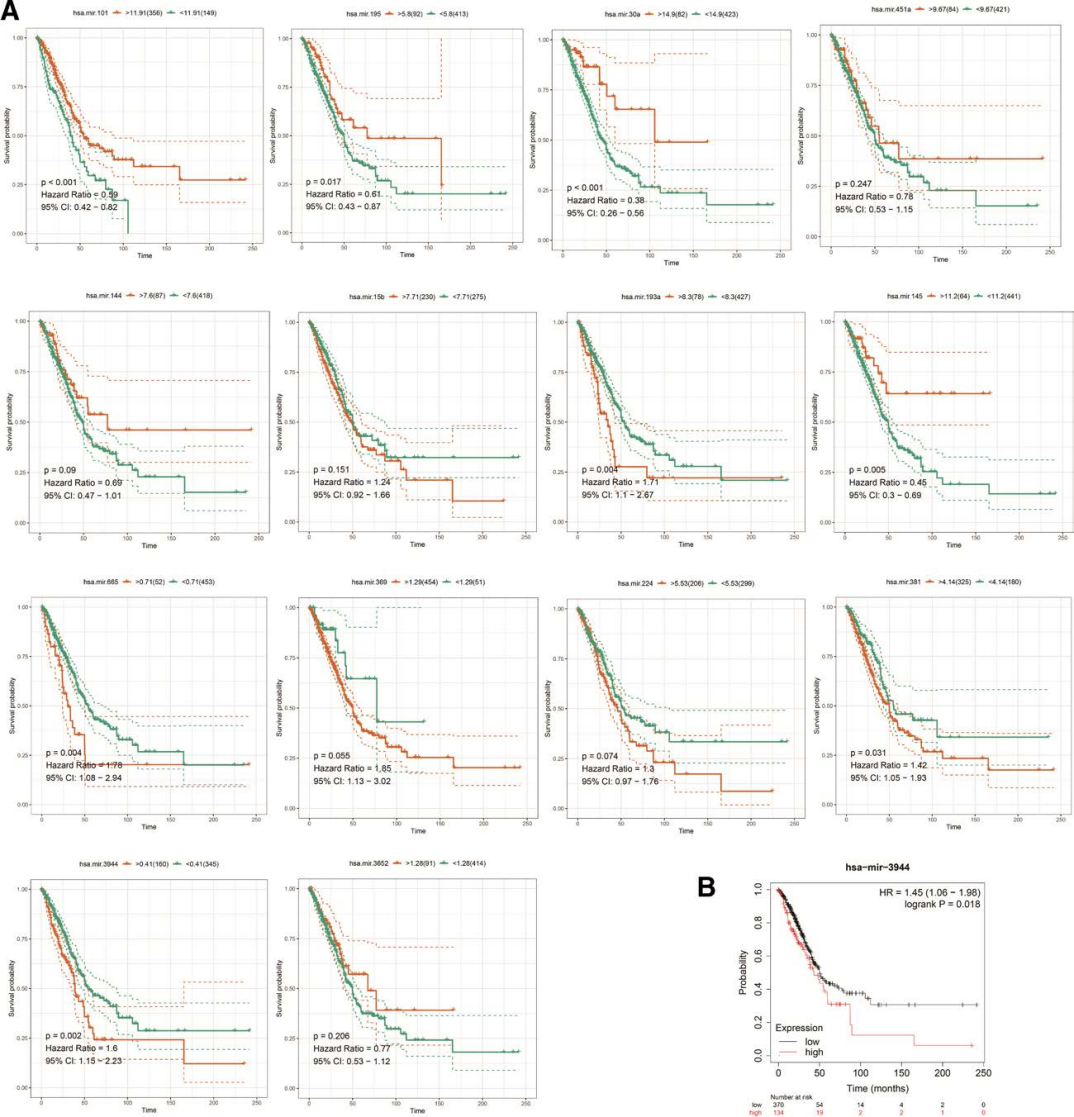
Overall survival analyses of DEMs. (A) Overall survival analysis of 14 DEMs using R packages. (B) Overall survival analysis of hsa-miR-3944 using Kaplan–Meier Plotter (http://kmplot.com/). DEMs = differentially expressed mRNAs.

We used the TCGA database and R package to evaluate the DEMis we selected. Our results showed that low expression level of hsa-miR-101, hsa-miR-195, hsa-miR-30a, hsa-miR-145 (*P* < .05) was associated with poor OS in LUAD (Fig. 7A). In contrast, high expression level of hsa-miR-665, hsa-miR-381, hsa-miR-3944 (*P* < .05) was also associated with poor OS in LUAD (Fig. 7A). Hsa-miR-3944 was highly expressed and the high expression level of hsa-miR-3944 was associated with poor OS in LUAD. Then we used Kaplan–Meier Plotter (http://kmplot.com/) to perform an OS analysis for hsa-miR-3944. As shown in the Figure 7B, the same result is displayed in the Kaplan–Meier Plotter.

## 4. Discussion

Early extensive metastasis and high degree of malignancy are the main characteristics of LUAD. The 2-year survival rate of patients with extensive disease is about 5%, and the survival time is only 8–13 months.^[30]^ Most patients have distant metastases and poor prognosis at the time of diagnosis.^[31]^ At the molecular level, the pathogenesis of LUAD is still unclear. Therefore, there is an urgent need to find more effective biomarkers for early diagnosis and treatment. Microarray technology is an effective method to analyze biomarkers and can be used to study the gene expression profile in LUAD.^[32]^ In addition, miRNA can affect the occurrence and metastasis of LUAD by downregulating or upregulating mRNA expression levels.^[33]^ In this study, the R platform was used to analyze the miRNAs, mRNAs, and lncRNAs in LUAD, and Cytoscape software was used to construct a ceRNA regulatory network to explore the molecular pathological mechanism of LUAD.^[34]^

We screened the miRNA and mRNA microarray data of 5 LUAD patients’ tumor tissues and adjacent normal tissues from the GEO database. The miRNA and mRNA data were screened from the same patients to better analyze and predict the relationship between miRNA and lung cancer. We evaluated the changes in miRNA and mRNA expression in LUAD by integrating miRNA and mRNA expression profiles. A total of 625 DEMis (272 upregulated miRNAs and 353 downregulated miRNAs) and 3135 DEMs (1659 upregulated mRNAs and 1476 downregulated mRNAs) were identified. These mRNAs and miRNAs significantly participate in 70 signaling pathways. Among them, Wnt signaling pathway, PI3K-Akt signaling pathway, and Notch signaling pathway are implicated in the occurrence of lung cancer.^[35–37]^ We wanted to evaluate these pathways in the selected cohort of 5 lung cancer patients. Then we listed the top 50 upregulated and top 50 downregulated miRNAs and validated them with the TCGA-LUAD database. A total of 14 miRNAs were found to also be differentially expressed in the TCGA-LUAD database, including 8 downregulated miRNAs and 6 upregulated miRNAs.

These 14 miRNAs may play a role in the pathogenesis of LUAD and have the potential to become biomarkers of LUAD. Among the 8 downregulated miRNAs, all have been reported to have reduced expression in cancer or have the function of suppressing tumors. Among the 6 upregulated miRNAs, 2 miRNAs have been reported to have elevated expression in cancer. The review of some relevant studies is presented in Table 4. These studies can provide references for us to screen and analyze miRNAs.

**Table 4:** The review of some relevant studies.

miRNA	Change	Tumor type	References	miRNA	Change	Tumor type	References
hsa-miR-101-3p	↓	NSCLC, BC, GC	^[38–40]^	hsa-miR-15b-5p	↓	NB	^[41]^
hsa-miR-195-5p	↓	NSCLC, EC, CC	^[42–44]^	hsa-miR-193a-3p	↓	G, CC	^[45,46]^
hsa-miR-30a-3p	↓	NSCLC, GC, HCC	^[47–49]^	hsa-miR-145-5p	↓	HCC, GC	^[50,51]^
hsa-miR-451a	↓	NSCLC, CC,	^[52,53]^	hsa-miR-665	↑	NSCLC, GC, BC	^[54–56]^
hsa-miR-144-3p	↓	NSCLC, GC, CC	^[57–59]^	hsa-miR-224-3p	↑	NSCLC	^[60]^

BC = breast cancer, CC = colorectal cancer, EC = esophageal cancer, G = glioma, GC = gastric cancer, HCC = hepatocellular carcinoma, NB = neuroblastoma, NSCLC = non–small cell lung cancer.

Next, we used Targetscan and MiRBase to predict the target genes of these 14 miRNAs.^[61]^ We chose 3 relevant signaling pathways in cancer development (Wnt, PI3K-Akt, and Notch), and scanned for proteins involved in them that are potential targets of the aforementioned miRNAs.

**Figure 8.**
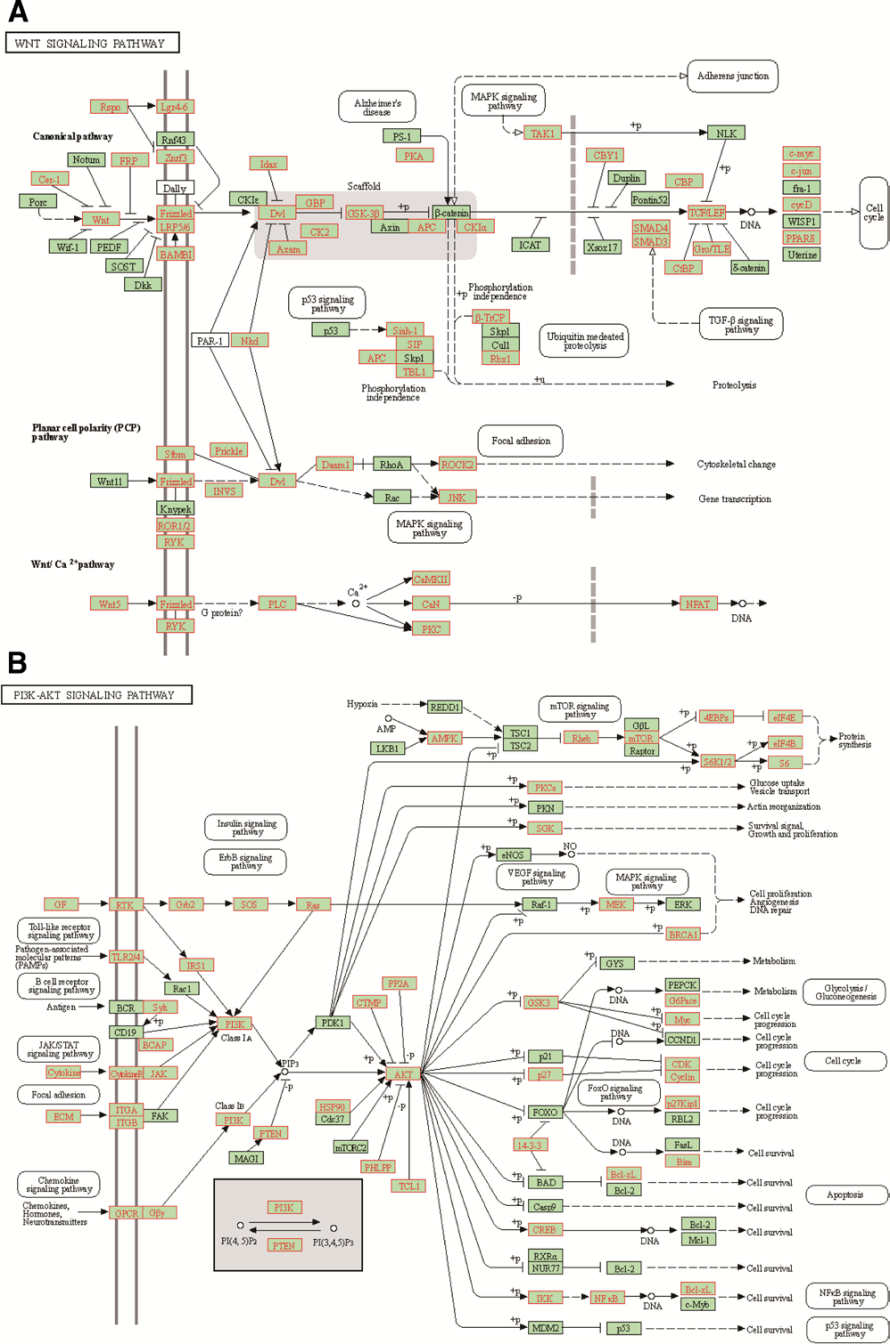
The target genes of hsa-miR-30a-3p in Wnt signaling pathway (A) and PI3K-Akt signaling pathway (B). The red genes in the figure are the target genes of hsa-miR-30a-3p.

Among the 8 downregulated miRNAs, the target genes of hsa-miR-30a-3p are abundant in the Wnt signaling pathway, including *WNT*, *FZD*, *DVL*, *LEF*, *CCND*, *PLC* and other family-related mRNAs, whose high expression can activate the Wnt signaling pathway to promote the occurrence of LUAD (Fig. 8A). The low expression of hsa-miR-30a-3p may lead to increased expression of these mRNAs, thereby promoting the activation of the Wnt signaling pathway. In addition, *FZD4* and *DVL1* are the target genes of hsa-miR-144-3p. *WNT2B*, *WNT3A*, *WNT4*, *WNT7A*, *FZD4*, *FZD6*, *CCND1*, *CCND2* and *CCND3* are the target genes of hsa-miR-195-5p and hsa-miR-15b-5p. *WNT2B*, *FZD4* and *CCND2* are the target genes of hsa-miR-145-5p. These genes can positively regulate the Wnt signaling pathway. Next, we analyzed the upregulated miRNAs. *SOST*, *SOX17*, and *NLK* are the target genes of hsa-miR-665. *NKD1*, *GSK3B*, and *TLE4* are the target genes of hsa-mir-369. *DKK1*, *SFRP1*, *SFRP2*, *CXXC4*, *GSK3B*, *CTBP1*, *CTBP2* are the target genes of hsa-mir-224. *SOST*, *DKK3*, *SFRP2*, *CXXC4*, *CTBP2*, and *NLK* are the target genes of hsa-mir-381. *SFRP5*, *NOTUM*, *NKD1*, *AXIN1*, *APC2*, *CTNNBIP1*, *SOX17*, *CTBP1* are the target genes of hsa-mir-3652. These mRNAs can negatively regulate the Wnt signaling pathway. The high expression of these miRNAs may lead to a decrease in the expression of these target genes and activate the Wnt signaling pathway.

We also analyzed the relationship between miRNAs (8 downregulated miRNAs and 6 upregulated miRNAs) and the PI3K-Akt signaling pathway. Hsa-miR-30a-3p can target *PI3K*, *AKT*, *MTOR*, and other important genes in the PI3K-Akt signaling pathway. Among the 8 downregulated miRNAs, hsa-miR-30a-3p has the most target genes in the PI3K-Akt signaling pathway (Fig. 8B). *PIK3CB*, *AKT3*, and *MTOR* are the target genes of hsa-miR-101-3p. *IRS1* and *PIK3CB* are the target genes of hsa-miR-144-3p. *IRS1*, *PIK3R1*, and *AKT3* are the target genes of hsa-miR-15b-5p and hsa-miR-195-5p. *IRS1* and *AKT3* are the target genes of hsa-miR-145-5p. These genes can activate the PI3K-Akt signaling pathway, and the low expression of these miRNAs may lead to the upregulation of these genes. Among the 6 upregulated miRNAs, we analyzed several key tumor suppressor genes. *PTEN* is the target gene of hsa-miR-224-3p and hsa-miR-369-3p.*TP53*, *MAGI*, and *PTEN* are the target genes of hsa-miR-3652. *MAGI* and *TSC1* are the target genes of hsa-miR-3944.The upregulation of these miRNAs may result in the loss of *TP53*, *MAGI*, *TSC1*, and *PTEN* expression.

Finally, we analyzed the relationship between miRNA and the Notch signaling pathway. *DLL1*, *DLL4*, *NOTCH2*, and *RBPJ* are the target genes of hsa-miR-15b-5p and hsa-miR-195-5p. The low expression of these 2 miRNAs may lead to the activation of the Notch signaling pathway.

In conclusion, through a bioinformatics approach, we searched for differentially expressed miRNAs, mRNAs, and lncRNAs in LUAD from selected GEO datasets. We corroborated DEMs in the TCGA database and used DEMs as the core to explain the possible relationship between DEMis and lung cancer, and the possible relationship between DEMis and upstream lncRNA. Then we used Targetscan and miRBase to predict target genes in 3 signaling pathway, PI3-Akt, Wnt, and Notch. In addition, we tried to create a regulatory network (ceRNA networks) of identified miRNAs, lncRNAs, and mRNAs in LUAD to identify potential biomarkers and possible targets for gene therapy of this disease. Some of these DEMis play a role in lung cancer or other cancers. The results of our analysis predicted potential mechanisms of these DEMis and identified which miRNAs were associated with poor OS in LUAD. Low expression level of hsa-miR-30a was highly associated with poor OS in LUAD (*P* < .001). Hsa-miR-30a-3p may suppress the occurrence and progression of lung cancer through Wnt and Akt signaling pathways and, as a result, could become a good biomarker in LUAD. In addition, our results reported for the first time that hsa-miR-3944 and hsa-miR-3652 are highly expressed in LUAD and we predicted their target genes. It makes more sense that the high expression level of hsa-miR-3944 is associated with poor OS. At present, hsa-miR-3944 has not been studied in the cancer field, and it has the potential to become a new LUAD biomarker. This research can provide a reference for screening biomarkers related to the diagnosis and treatment of lung cancer in the field of miRNA.

The 5 microarrays from LUAD patients which we have analyzed for differential expression and validated in TCGA database come from another study.^[62]^ Jiang et al used three independent cohorts from China for validation and identified miR-1275 as upregulated in primary LUAD. We chose to validate the same data expression sets using curated data from public TCGA-LUAD repository and obtained different results. Genetic differences in the validation groups could have influenced these results, as Jiang’s validation cohort was biased toward Asians, and TCGA is biased to white people, with 393 white samples, 53 black or African American, 8 Asian, and 67 unknown. Similarly, Wu et al analyzed the TCGA database and identified different miRNAs as being most involved in LUAD.^[63]^ In their analysis they excluded patients that received treatment, had other primary cancers or had unknown clinical data. On the contrary, we chose to select DEMIs from the primary LUAD tissues and then to validate them with heterogenous group from TCGA database, in order to try and capture the genes that are important in cell transformation, since they are present at the onset of disease, and are persistently changed across different groups of LUAD patients. Two miRNAs were identified as significantly decreased by both approaches, hsa-mir-144 and hsa-mir-195. Azuma et. al identified miR-564 or miR-658 as upregulated in cell line PS-9/ZD, which is derived from gefitinib resistant LUAD cells.^[64]^ Subsequent experiments and transfection of gefitinib sensitive cell line PC-9, with miR-564 or miR-658 conferred resistance to gefitinib and increased survival rates of PC-9 cells, suggesting the role for these miRNAs in resistance to chemotherapy. Our results should also be verified with further experiments, in order to decipher the roles of hsa-miR-3944 and hsa-miR-3652 in tumor ontology and prospective trials should be made to test whether they are reliable markers for early detection of LUAD.

However, this research still has some limitations. First, as mentioned in the above paragraph, genetic differences in the validation groups could have influenced these results. In addition, our results depend solely on bioinformatics for analysis, which needs to be further validated by biological experiments and clinical experiments. Going forward, researchers cannot blindly select RNAs for experiments. Because the amount of RNA is very large, it is impossible to perform biological experiments on all RNAs. So our bioinformatics analysis can provide direction, theoretical basis and evidence for further biological experiments, although bioinformatics analysis cannot replace biological experiments. Insights from bioinformatics analysis studies could open up a new era in lung cancer treatment and lay the groundwork for the development of new therapies and/or early-stage disease biomarkers.

## 5 Conclusion

Hsa-miR-30a-3p may suppress the occurrence and progression of lung cancer through Wnt and AKT signaling pathways and become a good biomarker in LUAD. Hsa-miR-3944 and hsa-miR-3652 may serve as new biomarkers in LUAD.

## Author contributions

Study design: Y.S.; data extraction and data analysis: Y.S. and L.K.; manuscript writing and edition: Y.S., L.K., L.Z., and I.K.; conceptualization: Y.S., I.K.; investigation: Y.S., L.K., I.K.; data curation, formal analysis, methodology, software: Y.S., I.K.; supervision: I.K.; validation: Y.S., L.K., I.K.; visualization: Y.S., L.K.; Writing—original draft: Y.S.; Writing—review & editing: Y.S., L.K., L.Z., I.K.

The authors have no funding and conflicts of interest to disclose.

## Supplementary Material


